# Supplementary material for: Analysis of PPARγ Signaling Activity in Psoriasis
Source: Int J Mol Sci. 2021 Aug 10;22(16):8603. doi: 10.3390/ijms22168603 (PMC8395241; doi:10.3390/ijms22168603)
Supplement: Supplementary file 1 [file ijms-22-08603-s001.zip › Supplemental materials_Analysis of PPARg signaling activity in psoriasis/Pathway models/Models images and html files/PPARG inhibits phenotypes assotiated with psoriasis/30489.html]

infection


# Disease infection

|  |  |
| --- | --- |
| URN | urn:agi-meshdis:Infection |
| Total Entities | 69 |
| Connectivity | 22575 |
| Name | infection |

---

|  |  |
| --- | --- |
| ChildConcepts | empyema |
|  | nosocomial infection |
|  | virus infection |
|  | abscess |
|  | prion disease |
|  | central nervous system infection |
|  | bacterial infection |
|  | sepsis |
|  | mycetoma |
|  | fungal infection |
|  | airborne infection |
|  | algal infection |
|  | application site infection |
|  | asymptomatic infection |
|  | peritoneal and retroperitoneal infection |
|  | intra-abdominal infection |
|  | bloodstream infection |
|  | bone and joint infection |
|  | infections associated with other infections or conditions |
|  | congenital infection |
|  | device infection |
|  | digestive system infection |
|  | head and neck infection |
|  | experimental infection |
|  | cardiovascular infection |
|  | urogenital infection |
|  | graft infection |
|  | hand infection |
|  | latent infection |
|  | infection complication |
|  | infection at the injection site |
|  | intrauterine infection |
|  | respiratory tract infection |
|  | lymphatic system infection |
|  | parasitic disease |
|  | nail infection |
|  | perinatal infection |
|  | persistent infection |
|  | primary infection |
|  | pseudoinfection |
|  | reinfection |
|  | tropical disease |
|  | zoonosis |
|  | healthcare-associated infection |
|  | communicable disease |
|  | waterborne disease |
|  | musculoskeletal infection |
|  | skin infection |
|  | sexually transmitted disease |
|  | cross-infection |
|  | focal infection |
|  | laboratory infection |
|  | puerperal infection |
|  | suppuration |
|  | prosthesis-related infection |
|  | tick-borne disease |
|  | community-acquired infection |
|  | soft tissue infection |
|  | pelvic infection |
|  | maternal infection |
|  | neonatal infection |
|  | anaerobic infection |
|  | rare infectious disease |
|  | febrile neutropenia |
|  | congenital enterovirus infection |
|  | congenital herpes virus infection |
|  | congenital Epstein-Barr virus infection |
|  | pneumonia caused by Pseudomonas aeruginosa infection |
|  | Mycobacterium xenopi infection |

---

|  |  |
| --- | --- |
| Pathway | Proteins with Altered Expression in Aging |
|  | Plasmin Effects in Inflammation |
|  | Airway Epithelial Cell Dysfunction |
|  | CFTR Related Ion-Channel Dysfunction in Cystic Fibrosis Airway Epithelium (Class III Mutations) |
|  | New Pathway (2) |
|  | PPARG inhibits phenotypes assotiated with psoriasis |
|  | efects selected |
|  | Neighbors of infection |
|  | 2\_Ion channel CFTR failure in cystic fibrosis airway epithelium (class III-IV mutations) |

---

|  |  |
| --- | --- |
| MedScan ID | 9003213 |

---

|  |  |
| --- | --- |
| Alias | autoinfections |
|  | Infection Unspecified |
|  | Infections |
|  | inapparent infections |
|  | Disease Due to Infections |
|  | inapparent infection |
|  | chronic infection |
|  | auto-infection |
|  | accidental infections |
|  | Infection |
|  | auto-infections |
|  | accidental infection |
|  | acute infection |
|  | simultaneous infection |
|  | autoinfection |
|  | Disease Due to Infection |
|  | infectious episodes |
|  | simultaneous infections |
|  | acute infections |
|  | chronic infections |
|  | infectivities |

---

|  |  |
| --- | --- |
| MeSH ID | D007239 |
|  | M0011294 |

---

|  |  |
| --- | --- |
| MeSH Heading | Infection |

---
